# P-1499. Patient Characteristics and Clinical Outcomes Associated with Meropenem/Vaborbactam Treatment in Carbapenem-Resistant *Enterobacterales* Pneumonia

**DOI:** 10.1093/ofid/ofae631.1668

**Published:** 2025-01-29

**Authors:** Kaylee E Caniff, Chloe Judd, Taryn A Eubank, Kevin W Garey, Tamara Krekel, Wesley D Kufel, Justin A Andrade, John Cerenzio, Michael Veve, Michael J Rybak

**Affiliations:** Anti-Infective Research Lab, Eugene Applebaum College of Pharmacy and Health Sciences, Wayne State University, Royal Oak, Michigan; Wayne State University, Detroit, Michigan; University of Houston College of Pharmacy, Houston, Texas; University of Houston, Houston, TX; Barnes-Jewish Hospital, St. Louis, MO; Binghamton University School of Pharmacy Sciences, Binghamton, NY; The Brooklyn Hospital Center, Brooklyn, New York; Brooklyn Hospital, Brooklyn, New York; Henry Ford Health, Detroit, Michigan; Eugene Applebaum College of Pharmacy and Health Sciences, Detroit, Michigan

## Abstract

**Background:**

Carbapenem-resistant *Enterobacterales* (CRE) are a major public health threat due to increasing prevalence and limited effective treatment options. Meropenem/vaborbactam (M/V) is a beta-lactam/beta-lactamase inhibitor agent designed to treat CRE. There is a paucity of data related to patient characteristics and associated outcomes with the utilization of M/V in pneumonia due to CRE. The objective of this study was to describe real-world experience with M/V in this setting.

**Figure:**
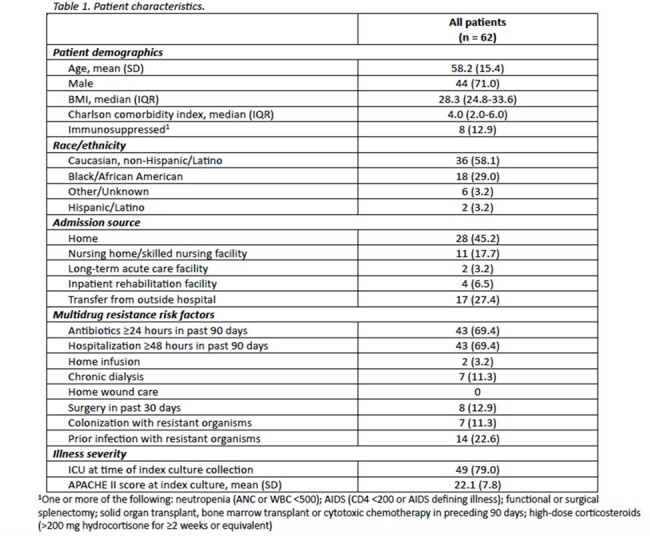

**Methods:**

This is a retrospective, observational, multicenter, cross-sectional study of patients ≥ 18 years old who received M/V for ≥ 72 hours for clinically-diagnosed hospital-acquired (HAP) or ventilator-associated pneumonia (VAP) due to CRE between 6/2020-2/2024. The primary outcome was clinical success, defined as resolution or improvement in signs/symptoms of infection and without the need for additional therapy. The secondary outcomes included 30-day mortality, 30-day microbiologic recurrence, 30-day symptomatic recurrence and adverse drug reaction(s).

**Figure:**
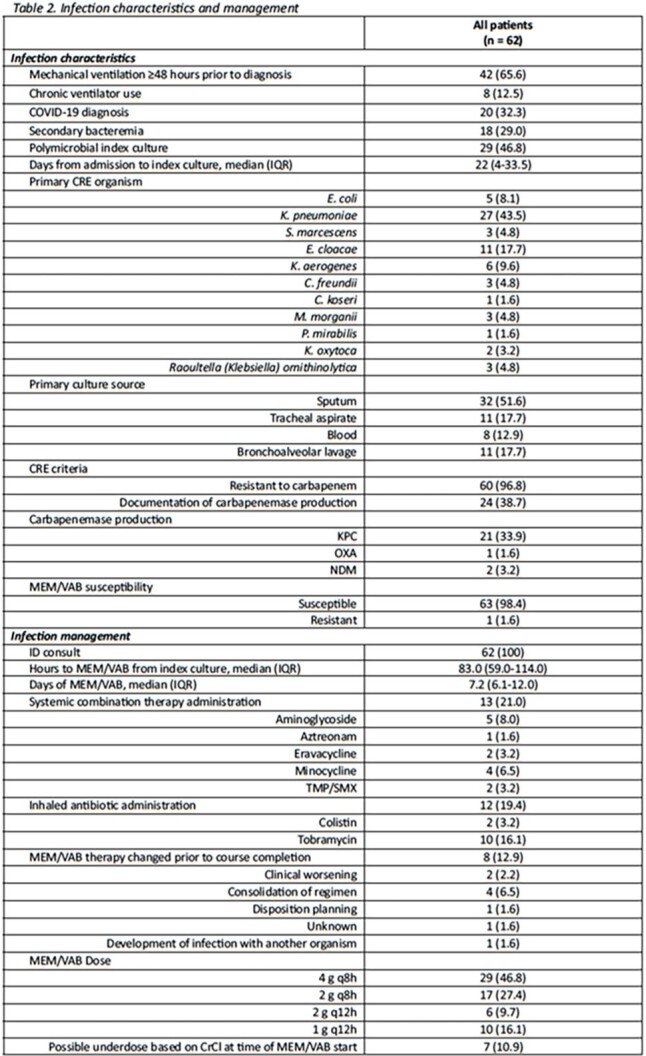

**Results:**

Sixty-two patients were included from six U.S. medical centers. The mean age (standard deviation [SD]) was 58.2 (15.4) years; patients were predominantly male (71.0%) and Caucasian (58.1%). At time of culture collection, the mean (SD) APACHE II score was 22.1 (7.8) and most patients (78.0%) were admitted to the intensive care unit. *Klebsiella pneumoniae* was the predominant species isolated in culture (43.5%). Notably, 65.6% were diagnosed with VAP, 32.3% had a COVID-19-related hospitalization and 29.0% developed secondary bacteremia. Patients received a median (interquartile range [IQR]) of 7.2 (6.1-12.0) days of M/V, with 21.0% receiving systemic combination therapy. Clinical success was achieved in 69.4% of patients and 30-day mortality occurred in 33.9%. Microbiologic and symptomatic recurrence occurred in 11.2% and 8.1% of cases, respectively. One patient experienced *Clostridioides difficile* infection attributed to the use of M/V.

**Figure:**
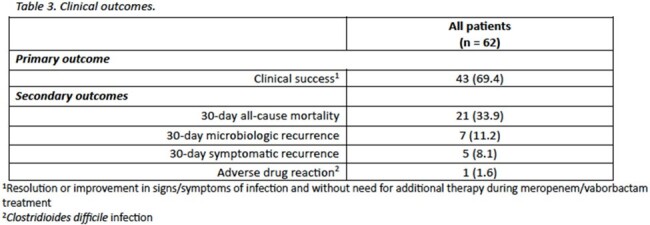

**Conclusion:**

Our study demonstrates promising clinical success with the use of M/V for treatment of pneumonia. Larger, comparative studies are needed in this population to identify patient factors associated with clinical success and assess M/V’s efficacy compared to other available therapies.

**Figure:**
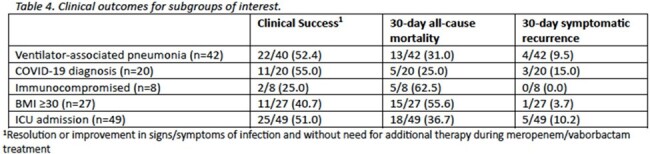

**Disclosures:**

**Kaylee E. Caniff, PharmD, BCIDP**, T2Biosystems: Honoraria **Kevin W. Garey, PharmD, MS**, Acurx: Grant/Research Support **Tamara Krekel, PharmD, BCPS, BCIDP**, Merck Inc: Honoraria **Wesley D. Kufel, Pharm.D., BCPS, BCIDP**, Merck & Co.: Grant/Research Support|Shionogi, Inc: Grant/Research Support **Michael J. Rybak, PharmD, PhD, MPH**, Abbvie, Melinta, Sionogi, Merck, T2Biosystems: Advisor/Consultant|Abbvie, Melinta, Sionogi, Merck, T2Biosystems: Grant/Research Support|Abbvie, Melinta, Sionogi, Merck, T2Biosystems: Speaker

